# Facile Synthesis of a 3,4-Ethylene-Dioxythiophene (EDOT) Derivative for Ease of Bio-Functionalization of the Conducting Polymer PEDOT

**DOI:** 10.3389/fchem.2019.00178

**Published:** 2019-03-29

**Authors:** Bingchen Wu, Bin Cao, Ian Mitch Taylor, Kevin Woeppel, Xinyan Tracy Cui

**Affiliations:** ^1^Department of Bioengineering, University of Pittsburgh, Pittsburgh, PA, United States; ^2^Center for the Neural Basis of Cognition, University of Pittsburgh, Pittsburgh, PA, United States; ^3^McGowan Institute for Regenerative Medicine, University of Pittsburgh, Pittsburgh, PA, United States

**Keywords:** thiol-ene, EDOT derivative, conducting polymer, bio-functionalization, organic electronics

## Abstract

In the pursuit of conducting polymer based bio-functional devices, a cost-effective and high yield synthesis method for a versatile monomer is desired. We report here a new synthesis strategy for a versatile monomer 2-methylene-2,3-dihydrothieno (3,4-b) (1,4) dioxine, or 3,4-ethylenedioxythiophene with a exomethylene side group (EDOT-EM). Compared to the previously reported synthesis route, the new strategy uses less steps, with faster reaction rate, and higher yield. The presence of EM group opens up endless possibility for derivatization via either hydro-alkoxy addition or thiol-ene click chemistry. EDOT-EM could be polymerized into stable and low impedance PEDOT-EM polymer using electro-polymerization method on different conducting substrates at both macro and micro scales. Facile post-functionalization of PEDOT-EM with molecules of varying size and functionality (from small molecules to DNAs and proteins) was achieved. The new synthetic route of EDOT-EM and the ease of post-functionalization of PEDOT-EM will greatly accelerate the use of conducting polymer in a broad range of organic electronics and bioelectronics applications.

## Introduction

Poly 3,4-ethylene dioxythiophene (PEDOT) is a widely used conductive polymer noted for its broad applicability, ranging from organic field-effect transistors, (Sirringhaus et al., 2000) solar cells, (Kim et al., 2011) and light emitting diodes (de Jong et al., 2000) to nanomedicine, (Karagkiozaki et al., 2013) biosensors and bioelectronics. (Cui and Martin, 2003; Martin, 2015) PEDOT have excellent conductivity, transparency, and processability, making it great candidates for fabrication of electrodes such as transparent organic solar cells or light emitting diodes (de Jong et al., 2000; Zhang et al., 2002; Sun et al., 2015). In particular, PEDOT has been widely utilized in the biomedical research front and is utilized for electrically controlled drug delivery, (Abidian et al., 2006; Luo and Cui, 2009; Chikar et al., 2012) conductive scaffolds for stimulation and tissue regeneration, (Bolin et al., 2009; Yazdimamaghani et al., 2015) and biosensors for chemical species such as dopamine, hydrogen peroxide, and acetylcholine (Istamboulie et al., 2010; Lin et al., 2010; Taylor et al., 2017b). Coating electrode with PEDOT is a commonly adopted strategy to enhance electrode performance due to high charge storage capacity, reversable redox activity, and extremely the high surface area of PEDOT films, which are highly desired properties for the applications of biosensing, recording, and stimulating devices (Luo et al., 2011; Lu et al., 2012; Taylor et al., 2017b). Within the contest of neural interfacing technology, PEDOT has been shown to be an excellent coating for neural electrodes due to its excellent biocompatibility and stability, and low impedance (Cui and Martin, 2003; Luo et al., 2008; Zhu et al., 2014; Kolarcik et al., 2015). Previous reports have demonstrated PEDOT based polymer coating can effectively improve sensitivity, stimulation and recording stability, and longevity of neural electrode *in vivo* (Schwartz et al., 2006; Luo et al., 2011; Du et al., 2015; Kolarcik et al., 2015; Vara and Collazos-Castro, 2015; Kozai et al., 2016; Taylor et al., 2017b).

In the pursuit of high efficiency and functional PEDOT based biomaterials and biomedical devices, it is highly desirable to incorporate readily available reactive groups in conducting polymers for surface modification and bioconjugation. Also, because of the requirement of tunability and processability in developing conducting polymers, electro-polymerization method has become a widely utilized convenient technique to precisely control polymerization process on conductive substrates. During the electro-polymerization, biomolecules may be incorporated in the polymer film as dopants, which is a convenient method for bio-functionalization (Stauffer and Cui, 2006; Boehler et al., 2017). The dopants are only physical entrapped in polymer film and can be released passively or actively, which is desirable in applications such as control drug delivery. However, there remains the need for a reliable and stable covalent functionalization method. Additionally, entrapped biomolecules have limited surface exposure, and if the bioactive sites of the molecule are blocked, the intended biological function will not be achieved. On the other hand, covalent attachment of biomolecules on the polymer surface can effectively overcome these limitations. Unfortunately, PEDOT lacks the necessary reactive sites for direct functionalization. This motivates the development of EDOT derivatives.

EDOT-OH is an EDOT derivative developed with the capability to undergo direct post-coating functionalization through reaction with a free hydroxymethyl group. EDOT-OH was first synthesized through the cyclization of diethyl 3,4-dihydroxythiophene-2,5-dicarboxylate through either a Williamson ether synthesis or Mitsunobu reaction pathway, followed by decarboxylation. This synthetic strategy was successful but resulted in poor overall yield. As a result, an alternative method was then developed to synthesize EDOT-OH from 3,4-dimethoxythiophene as the starting material, through an acid catalyzed transesterification pathway (Luo et al., 2008; Sekine et al., 2011). Electro-polymerization of PEDOT-OH and its application on neural electrode and biosensing were also demonstrated in literature (Xiao et al., 2006; Lu et al., 2012). However, the synthetic route of EDOT-OH involves complex synthesis and functionalization steps that are costly and with low yield. EDOT-acid is another EDOT derivative developed with the capability to covalently bind peptides on polymer surface. A number of researches have also showed successful functionalization of PEDOT-acid with biomolecules through EDC/NHS chemistry (Sirringhaus et al., 2000; Povlich et al., 2013). However, with the acid group, only a limited pool of molecules can be used for post-functionalization (Povlich et al., 2013). Another EDOT derivative EDOT-NH_2_ been used to enhance adhesion of polymer to substrate, but no bioconjugation via the amine group has been demonstrated (Ouyang et al., 2017). Recently, functionalization through thiol-ene click chemistry has become increasingly popular due to its versatility, fast reaction rate and high yield (Hoyle et al., 2004; Kade et al., 2010). Using this chemistry for immobilization of biomolecules such as amino acids, peptides, and proteins are especially attractive due to the mild reaction condition and prevalence of thiol group in biological molecules (Jones et al., 2009; Zhang et al., 2016). Thiol-ene chemistry has also been used to functionalize conducting polymer poly (3,4-propylenedioxythiophene) (ProDOT) with hydrophobic and hydrophilic functional groups via single- or double-ene side groups, demonstrating the versatile and facile tuning options, although no direct bioconjugations have been reported so far (Hoyle et al., 2004; Kade et al., 2010; Feldman and Martin, 2012; Wei et al., 2015). EDOT with a single ene, i.e., exomethylene side group, referred to as EDOT-EM, has been reported by Beverina and co-workers (Sassi et al., 2013) in 2013. The synthesis route reported was complicated with only an overall yield around 50%, preventing the wide spread use of EDOT-EM. Beverina et al. further showed that various EDOT derivatives could be produced from EDOT-EM through thiol-ene chemistry demonstrating the versatility of this monomer. However, in their work, electro-polymerization of EDOT-EM was not successful.

**Scheme 1:**
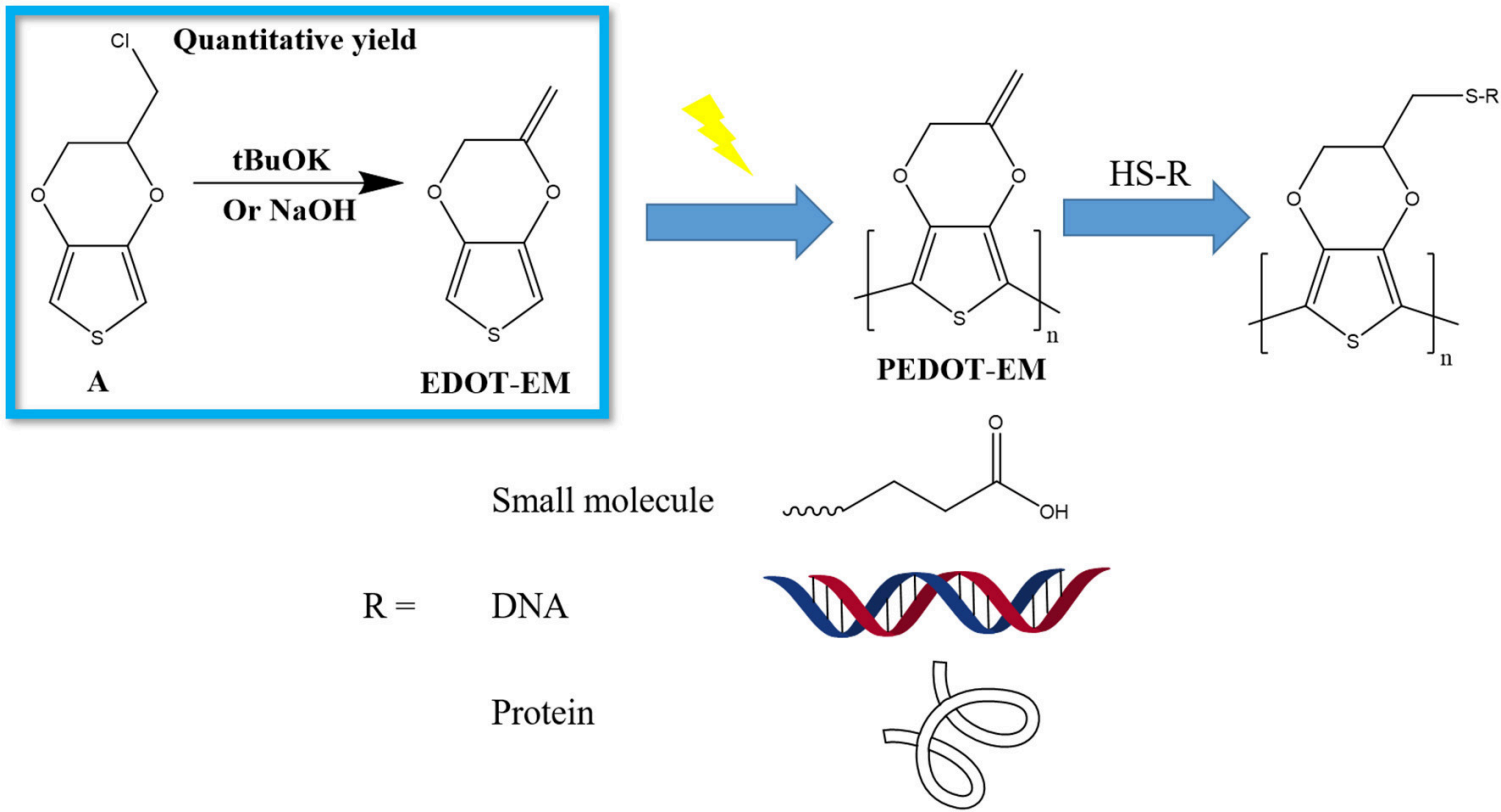
Schematic demonstration of the work flow to produce functionalized PEDOT-EM devices. **(A)** EDOT-MeCl.

In present work, we developed a novel synthesis route for EDOT-EM with one step, high conversion rate, and quantitative yield. We also optimized electro-polymerization method and produced stable and conductive PEDOT-EM film on macro and micro conductive surfaces. Furthermore, we demonstrated successful post-functionalization on PEDOT-EM polymer coatings with various molecules (Scheme 1).

## Materials and Methods

All reagents and solvents were used without further purification. 3,4-dimethoxythiophene was purchased from Matrix scientific. 3,4-Ethylenedioxythiophene (EDOT), 3-chloro-1,2-propanediol, p-toluene sulfonic acid monohydrate, toluene, tetrahydrofuran (THF), hexane, 3-mercaptopropionic acid (MPA), 2,2-Dimethoxy-2-phenylacetophenone (DMPA), Sodium Hydroxide (NaOH), Potassium hydroxide (KOH), Tris(2-carboxyethyl) phosphine hydrochloride (TCEP), Methanol (MeOH) and dichloromethane were purchased from Sigma. Potassium tert-butoxide (tBuOK) was purchased from ACROS. Ethanol (EtOH) was purchased through Fisher. Aptamer 5′-HS-(CH2)6-AGACAAGGAAAATCCTTCAATGAAGTGGGTCG-(CH2)7-MB-3′ with a 5′ thiol group as covalent linkage site and 3′ methylene blue (MB) as electrochemical reporter was purchased through Biosearch Technologies, Inc., Platinum/Iridium wires were purchased from A-M systems.

### Synthesis of Monomers

#### 2-Chloromethyl-2,3-dihydrothieno [3,4-b] [1,4] dioxine EDOT-MeCl

The monomer 2-Chloromethyl-2,3-dihydrothieno[3,4-b] [1,4] dioxine (EDOT-MeCl) was synthesized using a method similar to a published procedure (Segura et al., 2006). In brief, to a two-necked round bottom flask under nitrogen purge, 60 ml of dry toluene, 5 ml (39.5 mmol) of 3,4-dimethoxythiophene, 10 ml (120 mmol) of 3-chloro-1,2-propanediol, and 0.57 g (3 mmol) of *p*-toluene sulfonic acid monohydrate were added. Solution was heated at 90°C for 16 h with a reflux apparatus assembled on top. After this time, another 10 ml (120 mmol) of 3-chloro-1,2-propanediol was added, and the solution was heated at solvent, crude product was purified through gel. Chromatography (hexane/dichloromethane 4/1 v/v). The final product is a white solid.

#### 2-Methylene-2,3-Dihydrothieno [3,4-b] [1,4] Dioxine EDOT-EM

##### tBuOK condition

To a single-necked round bottom flask, 0.7 g (3.67 mmol) of EDOT-MeCl dissolved in 5 ml of THF and 0.824 g (7.34 mmol) of tBuOK dissolved in 5 ml of THF were added. Flask was sealed with rubber plug and solution was kept at room temperature for 30 min with stirring. After removing solvent, the crude product was purified through silica gel chromatography (hexane/dichloromethane 9/1 v/v). After evaporating the solvent with a rotovap, final product was obtained as colorless liquid. H^1^, NMR (CDCl_3_, 400 MHz): δ [ppm]: 6.43 (d, *J* = 3.6 Hz, 1 H), 6.39 (d, *J* = 3.6 Hz, 1 H), 4.76 (d, *J* = 2 Hz, 1 H), 4.49 (d, *J* = 0.4 Hz, 2 H), 4.42 (d, *J* = 2 Hz, 1 H). C^13^, NMR: δ = 149.57, 141.09, 140.63, 100.5, 99.92, 92.64, 77.39, 77.07, 76.75, 65.29 ppm.

##### Diisopropylamine condition

To a single-necked round bottom flask, 0.7 g (3.67 mmol) of EDOT-MeCl dissolved in 5 ml of MeOH and 1.03 ml (7.34 mmol) of diisopropylamine were added. Flask was sealed with rubber plug and solution was heated to 90°C and left overnight with stirring. After removing solvent, crude product was purified through silica gel chromatography (hexane/dichloromethane = 9/1). After evaporating the solvent with a rotovap, no viable amount product was collected.

##### NaOH/KOH condition

To a single-necked round bottom flask, 0.22 g (1.16 mmol) of EDOT-MeCl dissolved in 2 ml of MeOH and 100 mg (1.81 mmol) of KOH or 80 mg (2 mmol) of NaOH dissolved in 2 ml of MeOH were added. Flask was sealed with rubber plug and solution was heated to 90°C and left overnight with stirring. After removing solvent, crude product was purified through silica gel chromatography (hexane/dichloromethane = 9/1). After evaporating the solvent with a rotovap, the final product was obtained as colorless liquid (yield 92%).

### Characterization

NMR spectrum were measured on a Bruker ultra-shield 400 plus. ATR FT-IR spectra were measured using a Bruker Vertex-70LS spectrometer. SEM images were taken on a JSM 6335F SEM.

### Electrochemistry

#### Instrument and Setup

All electrochemical experiments were performed using an Autolab potentiostat (Metrohm) in a three-electrode cell (1 ml) consisting of a working electrode (Au sputtered plastic/Au coated Si wafer/Pt-Ir wire), a platinum counter electrode, and an Ag/AgCl reference electrode.

#### Electro-Polymerization for PEDOT-EM

A solution of water/acetonitrile 1:1 (volume ratio) with 100 mM of monomer and 100 mM of LiClO_4_ electrolyte was used as working solution. For galvanostatic (GS) method, a constant current ranging from 20 uA to 200 μA was applied and tested. The 200 μA conditions was determined as optimal current and utilized to coat Au sputter coated plastic with a surface area of 0.38 cm^2^ for 45, 90, 180, and 360 s and Au coated Si wafer with a surface area of 0.385 cm^2^ for 360 s. For potentiostatic (PS) method, a constant voltage of 1.1 V was applied with a charge cutoff at 0.009, 0.018, 0.036, and 0.072 C for Au-Si wafers with the same dimension, matching the amount of charge that was injected under optimal GS method. For coating Pt-Ir wires with a surface area of 0.00385cm^2^, a 2 μA current was applied for 120 s.

#### Aptamer Functionalization Detection

Square wave voltammetry was applied with a scan rate of 25 Hz from −0.1 to −0.6 V, with an amplitude of −0.025V and step size of −0.005V.

### Post-functionalization of PEDOT-EM

#### MPA Functionalization

All PEDOT-EM and PEDOT coated Au-Si wafers were rinsed with EtOH three times before reaction. 64 mg (0.25 mmol) of DMPA, and 437 μl (5 mmol) of MPA were added into 5 ml EtOH solution in a Petri dish. PEDOT-EM and PEDOT coated Au plastic substrates were then immersed into solution and the petri dish was covered with a quartz glass lid and placed under UV lamp (100 w, Series 1000 Omni Cure^TM^) for 1 h. All Samples were rinsed with EtOH three times after reaction to remove MPA residues.

#### Aptamer Functionalization

10 μL of 50 mM TCEP were first added into 5 μL of 100 μM aptamer solution, and solution was kept at room temperature for 30 min in order to reduce the disulfide bond. Then aptamer solution was diluted to 10 μM with PBS. 25 μL of diluted aptamer solution were dropped on PEDOT-EM and PEDOT coated Au plastic substrates, covered with aluminum foil, and left at room temperature overnight. PEDOT-EM coated wires were immersed into the solution left at room temperature overnight. All samples were rinsed with PBS 3 times and stored in PBS before characterization.

#### Protein Functionalization

100 μl of 0.04 g/ml laminin solution was dropped on PEDOT-EM and PEDOT films on Au-Si wafer and allow protein to bind for 2 h at ambient temperature. Sample were then washed with PBS and incubated for 30 min at 37°C. After incubation, half of the samples were washed by 1% tween 20 (1 to 100 in PBS) to remove physically adsorbed laminin protein and rinsed with PBS extensively (15 times per sample). All other samples were submerged in PBS during the washing procedure. PBS was drained before plating the cells.

### Cell Culture and Immunostaining

#### Elution Test in Fibroblast Culture

3T3 fibroblast cells were plated in a 96-well plate at a density of 10^4^ cells/well in 100 μL of culture medium—DMEM/F-12, HEPES medium (Gibco) supplemented with 10% Fetal Bovine Serum (FBS) (Gibco) and 1% PenStrep (Life Technologies)—for 24 hr before use. Polymer films were immersed in 2 ml serum free and phenol red free culture medium incubated at 37°C for 72 h, after which 10% FBS was added back to medium before extraction. Elution solutions were made by mixing extractions with phenol red free medium at volume/volume ratio of 25, 50, 75, and 100%. Plating medium was then replaced with elution solutions and cells were then incubated at 37°C for 24 h after which a standard XTT assay was performed to text cytotoxicity of the elution.

#### Primary Neuron Cell Assay

All procedures followed NIH and federal guidelines and were approved by the University of Pittsburgh, the Institutional Animal Care & Use Committee. Primary neurons were isolated from E18 rat fetus (Taconic). In brief, E18 pregnant rats were euthanized by CO_2_ and the rat pups extracted. Pup brains were removed, and cortices were dissociated by Trypsin/EDTA solution (0.05%) followed by mechanical trituration with a fire-polished Pasteur pipette. Dissociated cells were collected by centrifuge (200 g, 5 min) and resuspended in NeuroBasal media (Gibco) supplemented with 2% B27 (Gibco) 1% GlutiMax (Gibco) and 1% PenStrep (Life Technologies). Cells were counted by taking a small aliquot of cell suspension and mixing 1:1 in Trypan blue solution (0.4%, BioWhittaker), following which cells were plated at a density of 2.5 × 10^5^ cells/cm^2^.

#### Immunostaining Procedure

Cells were cultured for 48 h and fixed with 4% para-formaldehyde for 30 min. Membranes were permeabilized with 0.2% Triton-X in PBS/Goat Serum (5%, Gibco). Mouse beta-3-tubulin primary antibody (Invitrogen) was introduced at 1:1,000 in PBS/Goat Serum and incubated for 2 h. Following washing with PBS, AlexaFluor-488 Rat-Anti-Mouse secondary antibody was introduced at 1:1,000 in PBS/Goat Serum incubated at 37°C for 45 min. Samples were washed again with PBS, and nuclei were labeled by Hoechst stain (Invitrogen), mixed at 1:1,000 in PBS and incubated for 10 min at 37°C. After staining, cells were washed twice with PBS and imaged by a fluorescence microscope (Leica DMI4000b).

## Results and Discussion

### One Step Synthesis of Edot-Em With Quantitative Yield

**Scheme 2:**
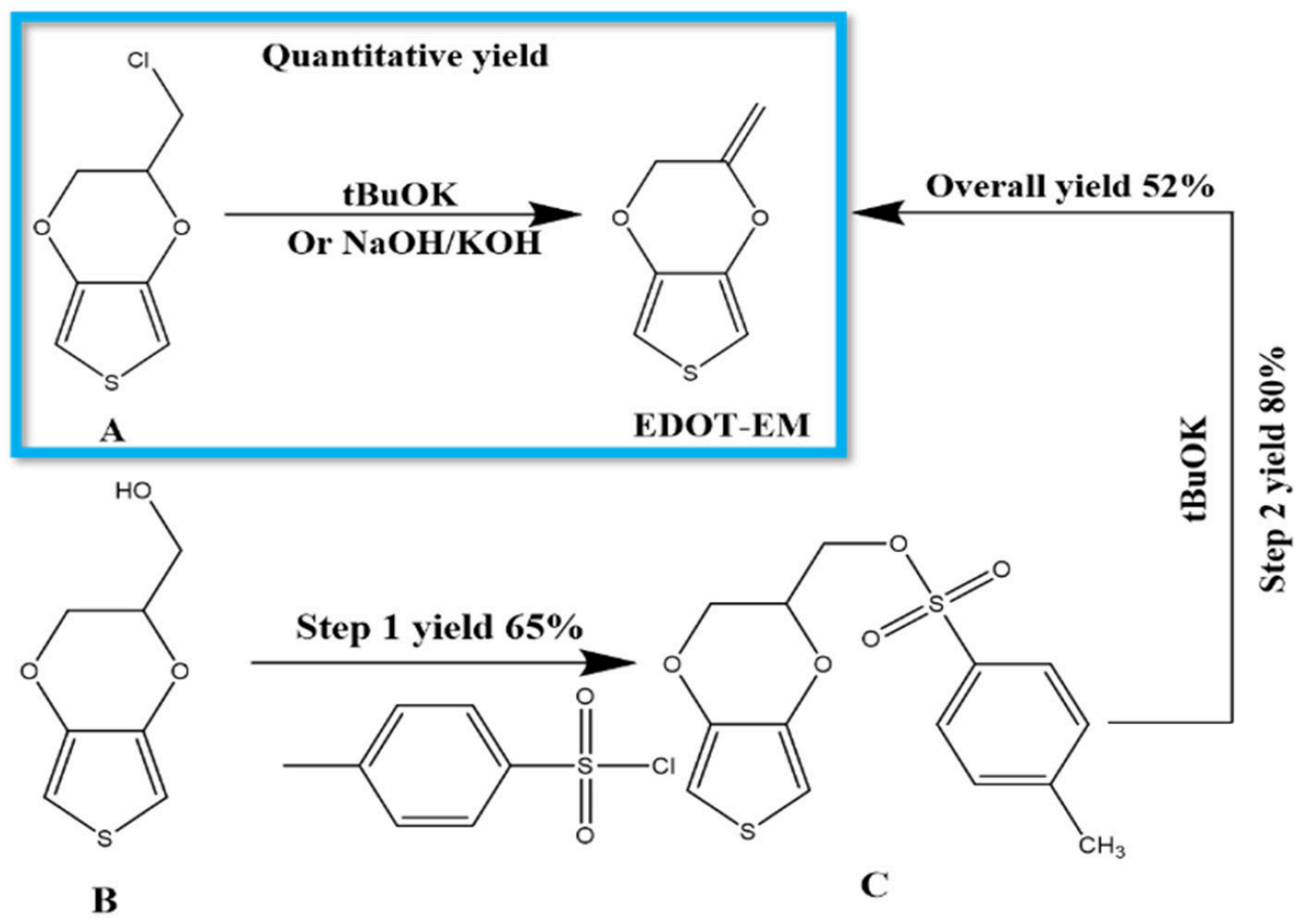
Synthesis of EDOT-EM. Our novel approach is highlighted in the blue box, which requires only 1 step with fast reaction rate, quantitative conversion, and extremely high yield, while the previously published route (outside of the box) showed more steps and lower yield (Sassi et al., 2013). **(A)** EDOT-MeCl, **(B)** EDOT-OH, **(C)** EDOT-Acid.

Previously EDOT-EM was synthesized using a 2 steps process by starting from EDOT-OH (Scheme 2) (Sassi et al., 2013). The first tosylation step of EDOT-OH has a 65% yield, which then followed by elimination of tosylate group with 80% yield. EDOT-EM was obtained with an overall yield around 52 % (Scheme 2). We made a discovery in the process of synthesizing EDOT-OH (compound **B**). During the reaction of EDOT-MeCl (compound **A**) with sodium acetate to make (2,3-Dihydrothieno[3,4-b] [1,4] dioxin-2-yl) methyl acetate (EDOT-Acid) at 120°C in DMSO, although the reactant was completely consumed, the conversion rate to targeting compound was found to be lower than expected. However, a side product was discovered with even lower polarity than the EDOT-MeCl. After isolation and purification, the obtained side product was examined by NMR spectrum and based on ^1^H, ^13^C spectra, and DEPT135 NMR spectrum (Figures S1, S2). We determined that the compound is a product from dehalogenation reaction upon heating at high temperature. Detailed atomic connectivity from two-dimensional NMR HSQC (Figure S3) and HMBC (Figure S4) confirmed the EDOT-EM structure. We propose that after elimination of HCl, the product converted into an EDOT-EM, and the newly-formed double bond could extend the conjugation thus stabilize the overall molecular structure.

Different reaction conditions were tested as shown in Table 1, three different types of reagents were tested to optimize the dehalogenation reaction conditions to produce EDOT-EM. Firstly, a non-nucleophilic strong base potassium tert-butoxide (tBuOK) was applied. Dehydrohalogenation was completed within 30 min with above 95 % isolation yield after washing and purification with silica gel chromatography. Secondly, reactions were attempted with milder bases either sodium hydroxide or potassium hydroxide. Conversion rate was slow at room temperature but could be completed upon heating at 90°C in a sealed reaction vessel overnight with a yield of 92%. This slightly lower yield was probably due to small amount of oligomerization or polymerization of EDOT-EM during heating. Lastly, a weak non-nucleophilic base, diisopropylamine, was tested at both room temperature and heated condition. Reaction conversion was very low, and no product was isolated. In conclusion, using this dehalogenation route and strong bases, the synthesis of EDOT-EM can be completed in one single step from EDOT-MeCl. Compared with the previously reported route for EDOT-EM synthesis, our approach takes fewer steps, with faster reaction rate, quantitative conversion, and quantitative yield. The presence of EM group opens up endless possibility for derivatization by either hydro-alkoxy addition or thiol-ene click chemistry (Sassi et al., 2013). In the previous report, (Sassi et al., 2013) it was demonstrated that EDOT-EM monomer can be functionalized and produce variety of monomer derivatives. Some of these derivatives, however, may not be amenable for electro-polymerization. The alternative might be more attractive and practical, in which EDOT-EM can be polymerized first and then undergoes post-functionalization via the ene group. Theoretically any molecules in the “tool-box” equipped with free thiol (SH) units could be directly attached to the polymer PEDOT-EM through the click chemistry.

**Table 1:** Reaction conditions tested to synthesize EDOT-EM.

**Reagent**	**Reaction Time**	**Solvent**	**Temperature**	**Yield**
tBuOK	30 min	Dry THF	RT	>95%
NaOH/KOH	Overnight	Methanol	90°C (sealed)	92%
Diisopropylamine	Overnight	Methanol	90°C (sealed)	–

### Electro-Polymerization of PEDOT-EM

**Figure 1:**
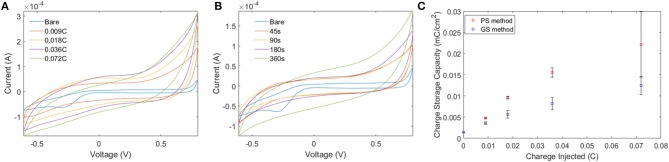
**(A,B)** Comparison of CV of PEDOT-EM acquired by GS method **(A)** and PS method. **(C)** Quantification of CSC for PS and GS methods. Both methods showed increased charge storage capacity proportional to amount of charge injected, with PS producing higher CSC than GS.

**Figure 2:**
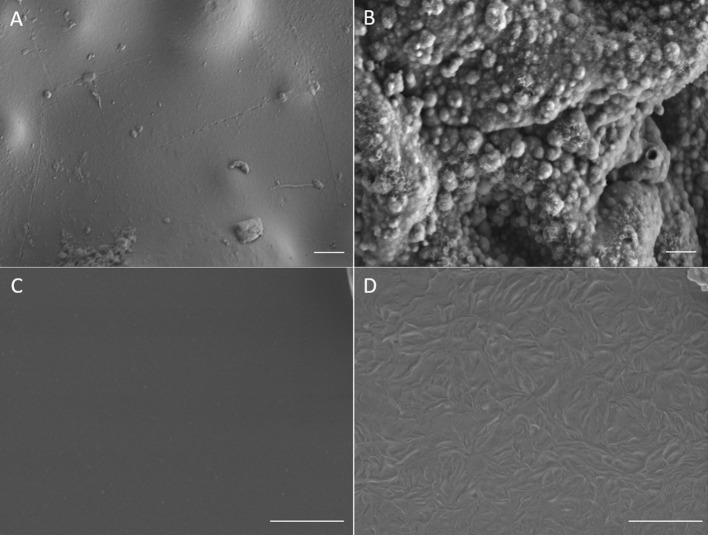
SEM images of **(A)** Bare gold surface on gold plastic electrode, **(B)** PEDOT-EM film coated gold plastic electrode surface (deposition charge 249.4 mC/cm^2^) **(C)**, Bare Pt/Ir wire and **(D)**, Pt/Ir wire coated with thin layer of PEDOT-EM (deposition charge 62.3 mC/cm^2^). Scale bar: 1μm.

Electro-polymerization method was employed to deposit conjugated PEDOT-EM onto different surfaces, such as iridium-platinum microwire (Pt-Ir), gold coated silicon wafers (Au-Si), and gold coated plastic. The electro-polymerization process could be monitored and processed in a precisely controlled manner by simply adjusting the potential/current and polymerization time (Cui and Martin, 2003). Previous work by Sassi et. al. only tested cyclic voltammetry method, which didn't successfully produce the polymer PEDOT-EM (Sassi et al., 2013). With the large quantity and high purity of EDOT-EM easily yielded using our new synthesis route, we were able to extensively test and optimize the electro-polymerization protocol. As indicated by previous research that deposition method affects polymer film properties, two different electrochemical methods were tested including the GS method that supplies a constant current and PS method that supplies a constant voltage (Cui and Martin, 2003). The amount of charge injected in both electro-polymerization methods were controlled and matched. The cyclic voltammetry of PEDOT-EM made by both methods were compared and the charge storage capacity (CSC) was calculated for each condition (Figure 1). Under both methods, the acquired PEDOT-EM films showed increased CSC proportional to the amount the charge that was injected (Figure 1C). Interestingly, although the total amount of charge injected in GS and PS method was matched, the CSC of polymer films are different. At the deposition charges of 0.018, 0.036 and 0.072 C, the CSCs of PEDOT-EM produced via the PS method are 9.6 ± 0.32, 15.5 ± 1.1, and 22.2 ± 7.7 mC/cm^2^, respectively, which are higher than the CSCs of polymer produced via GS method (5.7 ± 0.89, 8.2 ± 1.4, and 12.4 ± 2.1 mC/cm^2^, respectively). Such difference might be a result of different electro-polymerization voltages between the two methods. Under the GS method the voltage initially increased to around 0.9 V then stabilized around 0.85 V (data not shown), which is lower than the constant 1.1 V voltage applied under PS method. For the remaining experiments EDOT-EM were polymerized using GS method since GS provided a more stable and reliable polymerization control and previous research also indicated GS method produced more homogenous polymer film (Cui and Martin, 2003). We then examined the surface morphology of the PEDOT-EM polymer film grown on different substrates at different deposition charges by SEM (Figure 2). A rougher PEDOT-EM film with a cauliflower morphology typical of electrodeposited conducting polymer (Figure 2B) was produced comparing to bare gold surface (Figure 2A). On the other hand, a smoother surface was observed on PEDOT-EM coated Pt/Ir wires, where a thin film of PEDOT-EM produced wavelike structure features (Figure 2D). Minimum surface morphology changes were observed comparing to bare Pt/Ir wires (Figure 2C).

**Figure 3:**
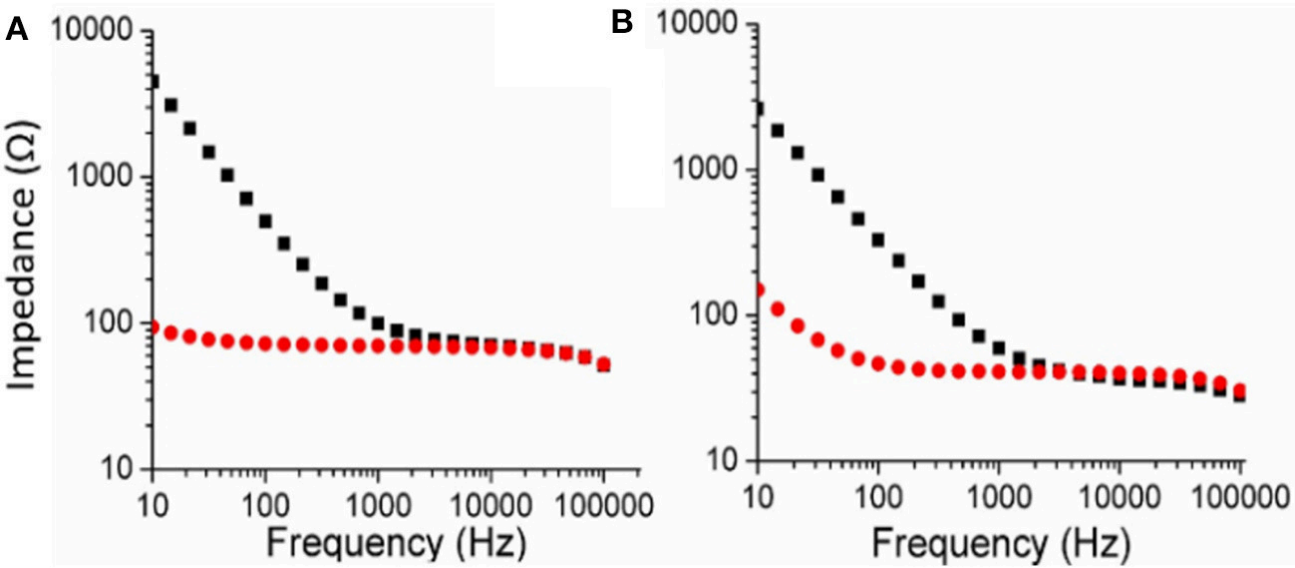
Comparison of EIS (Bode plots) for PEDOT **(A)** and PEDOT-EM **(B)** samples. EIS of bare Au-Si substrate was shown in black squares and polymer coated Au-Si substrate was shown in red dots. Deposition charge for both coating was 249.4 mC/cm^2^.

The Electrochemical Impedance Spectroscopy (EIS) of both PEDOT-EM and PEDOT polymerized on Au-Si wafers were characterized and compared (Figure 3). The impedance of the PEDOT-EM coated substrate was about over one order of magnitude lower than that of the uncoated gold at a broad range of low frequencies (Figure 3B), which can be explained by the increased surface area (Figure 2B). The reduction of impedance is comparable to that of PEDOT films (Figure 3A). Low impedance of PEDOT film has been shown to significantly improve microelectrode performances by decreasing recording noise and increasing charge injection limit (Cui and Martin, 2003; Abidian and Martin, 2008; Abidian et al., 2010; Harris et al., 2013; Castagnola et al., 2015; Du et al., 2015; Kolarcik et al., 2015; Charkhkar et al., 2016; Kozai et al., 2016). The EIS result indicates that the PEDOT-EM can exhibit similar impedance benefit as PEDOT, which is highly desired for applications such as neural electrode coatings.

### PEDOT-EM Post-functionalization

**Figure 4:**
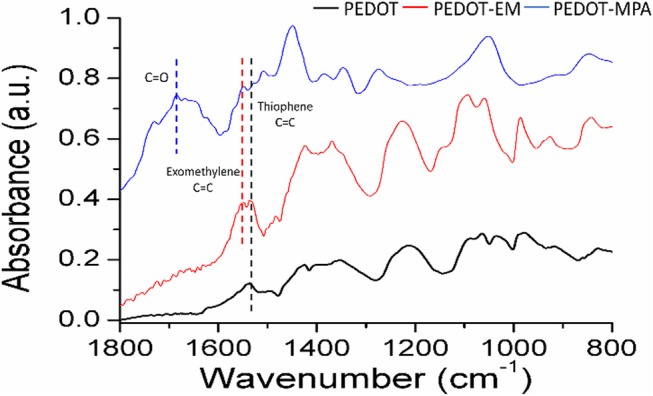
FTIR spectra comparison of PEDOT film treated with MPA, PEDOT-EM film before, and after functionalized with MPA. All films were obtained from electrochemical polymerization on Au-Si. Deposition charge for both coating was 249.4 mC/cm^2^.

To investigate whether the exomethylene group on PEDOT-EM was preserved after electro-polymerization process and can be further used for post-functionalization, we attempted to covalently attach molecules of different sizes to PEDOT-EM. We first post-functionalized the PEDOT-EM film with a small molecule, 3-mercaptopropionic acid (MPA) with the aid of a photo initiator (DMPA). Attenuated Total Reflectance-Fourier Transform Infrared (ATR FT-IR) spectroscopy was taken before and after functionalization of the PEDOT-EM films. For comparison, a PEDOT film was also treated with MPA and characterized with FTIR. The FTIR spectrum of the MPA treated PEDOT and PEDOT-EM slightly differs from each other. For PEDOT-EM, a peak at 1549 cm^−1^ can be attributed to the EM group, which was not seen in the spectrum of PEDOT. After thiol-ene functionalization of PEDOT-EM with MPA, appearance of the peaks of carbonyl C = O stretching, between 1600 and 1800 cm^−1^, and decreased signal intensity of EM group at 1,549 cm^−1^ confirmed the successful post-functionalization (Figure 4).

**Figure 5:**
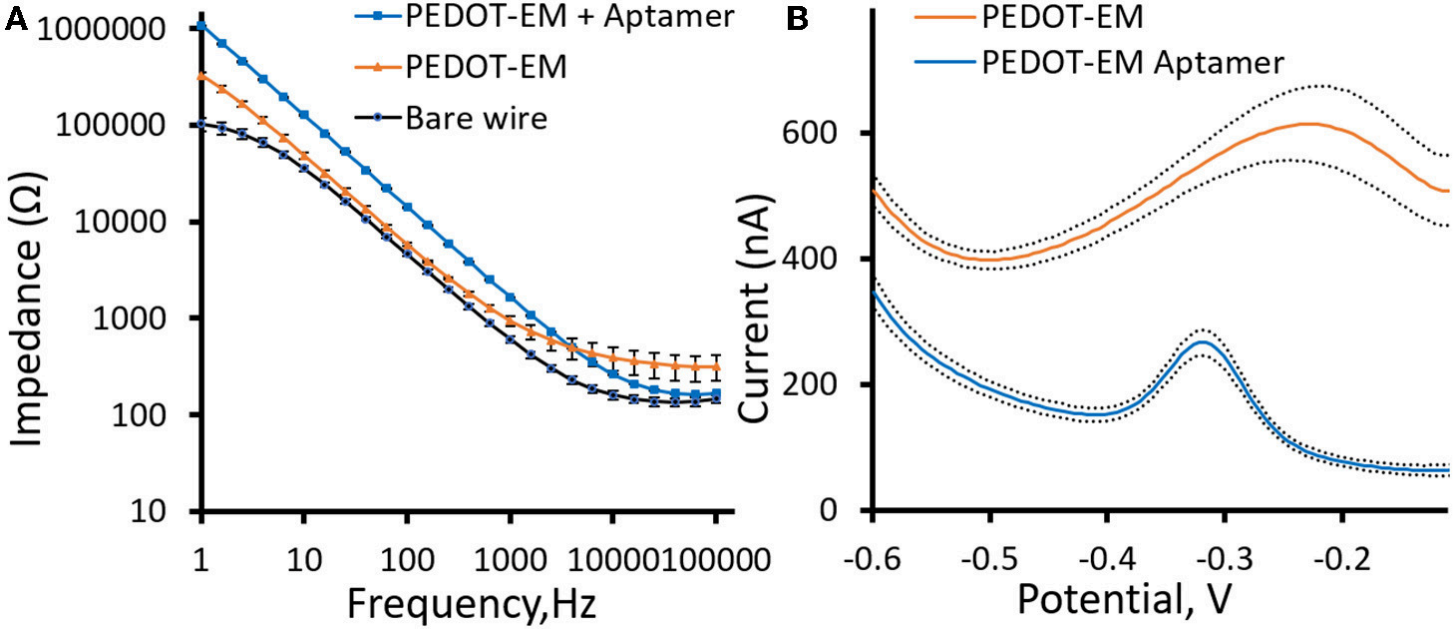
Aptamer functionalization **(A)**, Comparison of electrochemical impedance spectra (Bode plots) for PEDOT-EM functionalized with aptamer, PEDOT-EM, and bare Pt-Ir wires. **(B)**, Comparison of Square wave voltammetry scan before and after post-functionalization of PEDOT-EM, *n* = 5, Mean ± standard error.

For biosensors and bioelectrode applications, surface modifications with functional biological molecules that can recognize specific analytes are highly desirable. Pt-Ir microwires were coated with a thin layer of PEDOT-EM and post-functionalized with a DNA aptamer previously used for cocaine sensing (Baker et al., 2006; Taylor et al., 2017a). The aptamer contains a thiol group on 5′ that can be used as anchoring site and a methylene-blue group on 3′ that serves as electrochemical reporters. The impedance of Pt-Ir wires slightly increased with the deposition of a thin layer PEDOT-EM coating (Figure 5A). Here the polymerization condition was adjusted to obtain thin PEDOT-EM film with minimum surface area increase (Figure 2D) and impedance reduction, with the goal of minimizing background current in voltammetry sensing. Impedance further increased after aptamers were attached to PEDOT-EM polymer film, which is to be expected when nonconductive molecules are added to the electrode surface. Square wave voltammetry (SWV) was taken before and after post-functionalization of PEDOT-EM to detect successful immobilization of aptamer (Figure 5B). A methylene blue oxidation peak was clearly observed after PEDOT-EM was functionalized with aptamer, while no peaks were observed at the same voltage range for unfunctionalized PEDOT-EM film (Figure 5B). The observed peak position is consistent with previous literatures utilizing the same type of aptamers (Baker et al., 2006; Taylor et al., 2017a). Overall, PEDOT-EM functionalized with aptamer has a lower background current than PEDOT-EM alone. This observation is consistent with the EIS data, where PEDOT-EM has lower impedance than aptamer functionalized PEDOT-EM (Figure 5A). Additionally, FTIR spectroscopy verified the presence of aptamer on PEDOT-EM, while the PEDOT control showed minimum physical adsorption of aptamers (Figure S5). Together, the observed clearly resolved reduction peak at −0.3 V of methylene blue, the increased impedance after aptamer immobilization, and FTIR spectrum confirmed that the aptamer was successfully immobilized on the PEDOT-EM polymer surface. This opens the door for various aptamer based biosensing.

**Figure 6:**
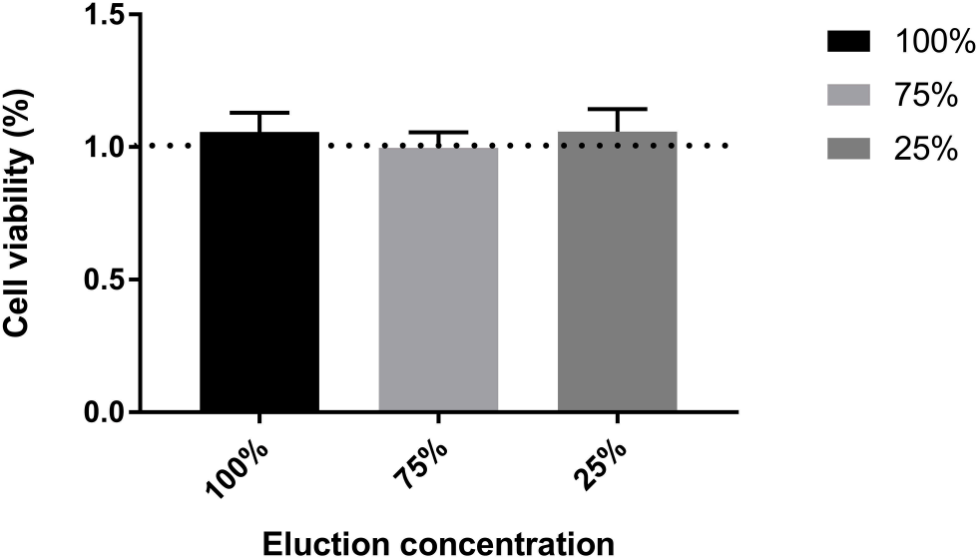
Cell viability test through XTT assay for different elution concentration. *n* = 12, no significant difference was found between groups, One-wat ANOVA, *p* = 0.7944.

For PEDOT-EM to be utilized in biomedical devices, it is important to confirm that it is non-toxic to human body. Cytotoxicity of PEDOT-EM was tested by the elution test followed by a standard XTT assay that tests the viability of cells using 3T3 fibroblast cells (Figure 6). No loss in cell viability was observed for cells grown in different concentrations of eluted solutions up to 100%. All experimental groups resulted in a cell viability around 100%, confirming PEDOT-EM is non-cytotoxic and safe to be utilized in biological applications.

**Figure 7:**
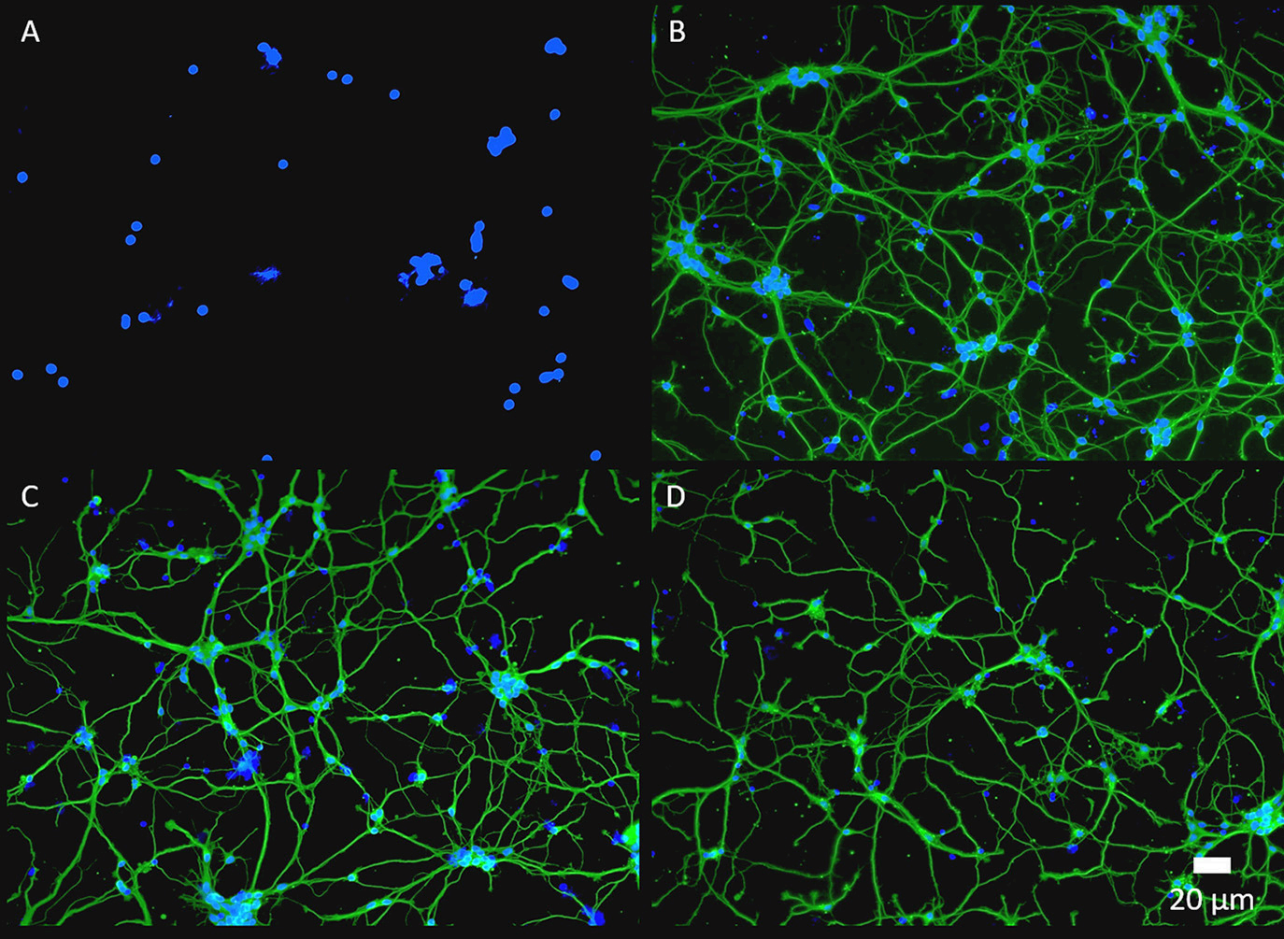
Fluorescence images of primary neuron culture on different surfaces. **(A)**, PEDOT-EM. **(B)**, NC-Laminin. **(C)**, PEDOT-EM-Laminin without tween-20 wash. **(D)**, PEDOT-EM-Laminin after tween-20 wash. ß tubulin (green) and cell nuclei (blue).

Often in biomedical applications, interactions between biological tissue and biomaterial surfaces need to be tailored based on the specific application. Peptides and proteins are commonly utilized biomolecules that serve as ligands and adhesion molecules to modulate device-tissue interactions. These biomolecules need to be attached to the biomaterial surface robustly to maintain device functionality and promote device integration within human body (Lauer et al., 2001; Luo and Shoichet, 2004; Azemi et al., 2008; Xu et al., 2009; Cheong et al., 2014; Hassarati et al., 2016). For this purpose, we further demonstrate the feasibility of post-functionalizing PEDOT-EM with larger biomolecule such as proteins. Laminin, an extracellular matrix protein known to promote neuron attachment and neurite outgrowth (Lander et al., 1985), were added to PEDOT and PEDOT-EM surfaces respectively and allowed to react with the surface for 2 h followed by PBS washes. Both surfaces were then treated with Tween 20 to remove the physically adsorbed laminin. FTIR spectroscopy showed that laminin on PEDOT was successfully removed by Tween 20 wash because the protein was physically adsorbed (Figure S6A) (Nosworthy et al., 2009). On the other hand, PEDOT-EM surface showed sustained and strong laminin signal after Tween 20 wash indicating a covalent binding (Figure S6B). Laminin functionalized PEDOT-EM (Figure 7C) shows neuron cell attachment and neurite outgrowth as good as the laminin coated nitrocellulose (a standard neural adhesive substrate for primary neuron culture) (Figure 7B), whereas PEDOT-EM alone does not support neuron attachment and growth due to lack of adhesion sites (Figure 7A). The laminin functionalized PEDOT-EM surface after Tween 20 wash (Figure 7D) showed similar neuronal growth as the surfaces without Tween 20 wash (Figure 7C), which confirms that laminin is firmly attached to the PEDOT-EM with covalent binding and remains bioactive.

## Conclusion

In conclusion, we have developed a novel approach to synthesize the versatile EDOT-EM, with fewer steps than the previously reported method, fast and quantitative conversion, and extremely high yield. EDOT-EM can be facilely and reproducibly polymerized into PEDOT-EM through electro-polymerization. The resulting PEDOT-EM films exhibit comparable conductivity to PEDOT, no cytotoxicity, and the capability of post-functionalization with small molecules, DNA aptamers, and large proteins. This monomer shows great promises for applications in organic electronics and bioelectronics. The ease of synthesis, polymerization and post-functionalization are expected to greatly accelerate the research and development of PEDOT based bio-functional devices in a broad range of applications.

## Data Availability

The raw datasets for this study will be available upon request.

## Author Contributions

BW designed the experiments, collected and analyzed all data, drafted up manuscript, and led the revision of manuscript. BC designed the experiments, collected preliminary data and participated in drafting manuscript. IT involved in polymerization condition optimization and aptamer sensor. KW involved in neuron cell culture experiments. PI XC supervised the entire project and edited the draft.

### Conflict of Interest Statement

The authors declare that the research was conducted in the absence of any commercial or financial relationships that could be construed as a potential conflict of interest.
