# Supplementary material for: Facile Synthesis of a 3,4-Ethylene-Dioxythiophene (EDOT) Derivative for Ease of Bio-Functionalization of the Conducting Polymer PEDOT
Source: Front Chem. 2019 Mar 29;7:178. doi: 10.3389/fchem.2019.00178 (PMC6450363; doi:10.3389/fchem.2019.00178)
Supplement: Supplementary file 1 [file Data_Sheet_1.PDF]

*Supplementary Material*

# Facile Synthesis of a 3,4-Ethylene-Dioxythiophene (EDOT) Derivative for Ease of Bio-functionalization of the Conducting Polymer PEDOT

**Bingchen Wu,[1,2‡] Bin Cao,[1‡] I. Mitch Taylor,[1,2] Kevin Woeppel,[1,2] and X. Tracy Cui.[1,2,3,*]**

*** Correspondence** X. Tracy Cui: xic11@pitt.edu

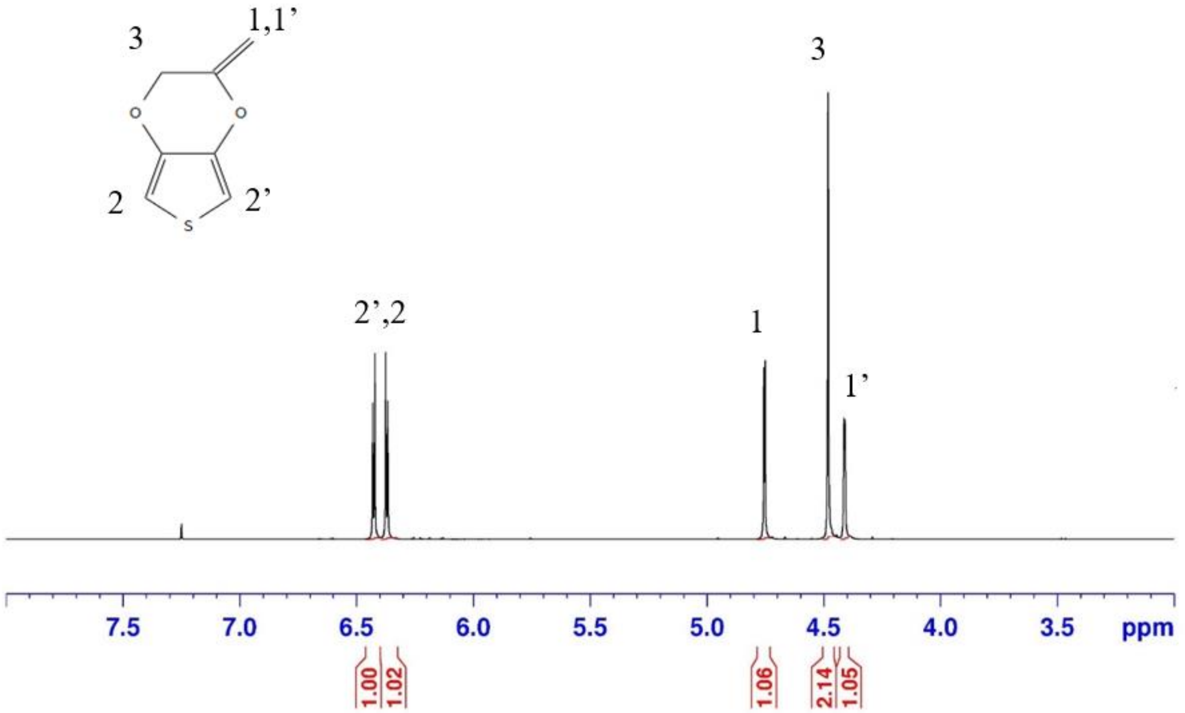


**Figure S1**. 1H NMR spectrum of EDOT-EM

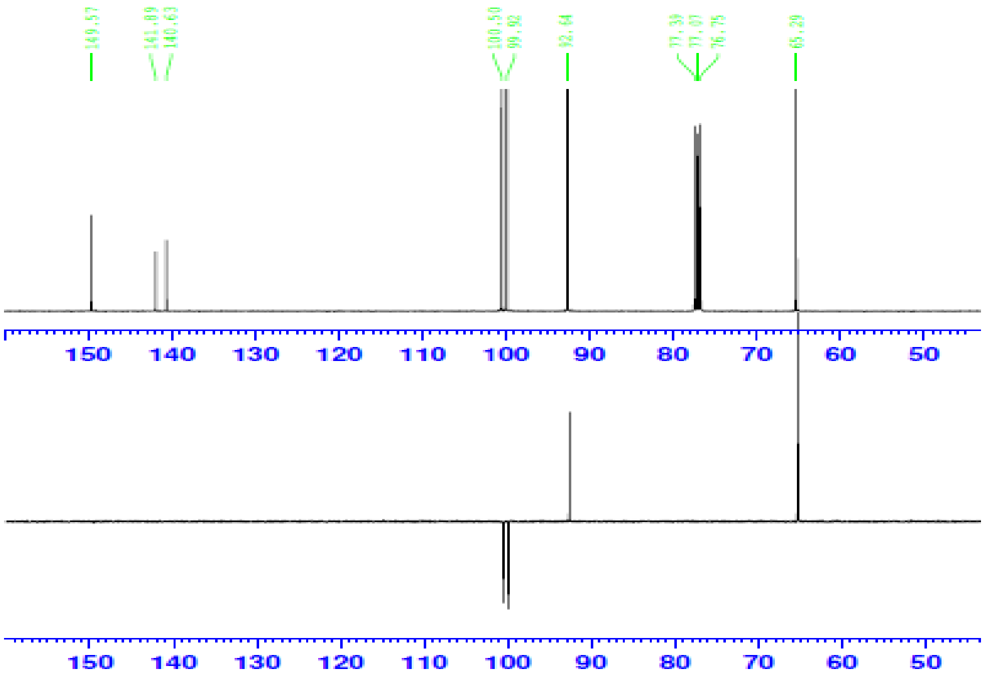


**Figure S2**. 13C (top) and DEPT135 (bottom) NMR spectra of EDOT-EM

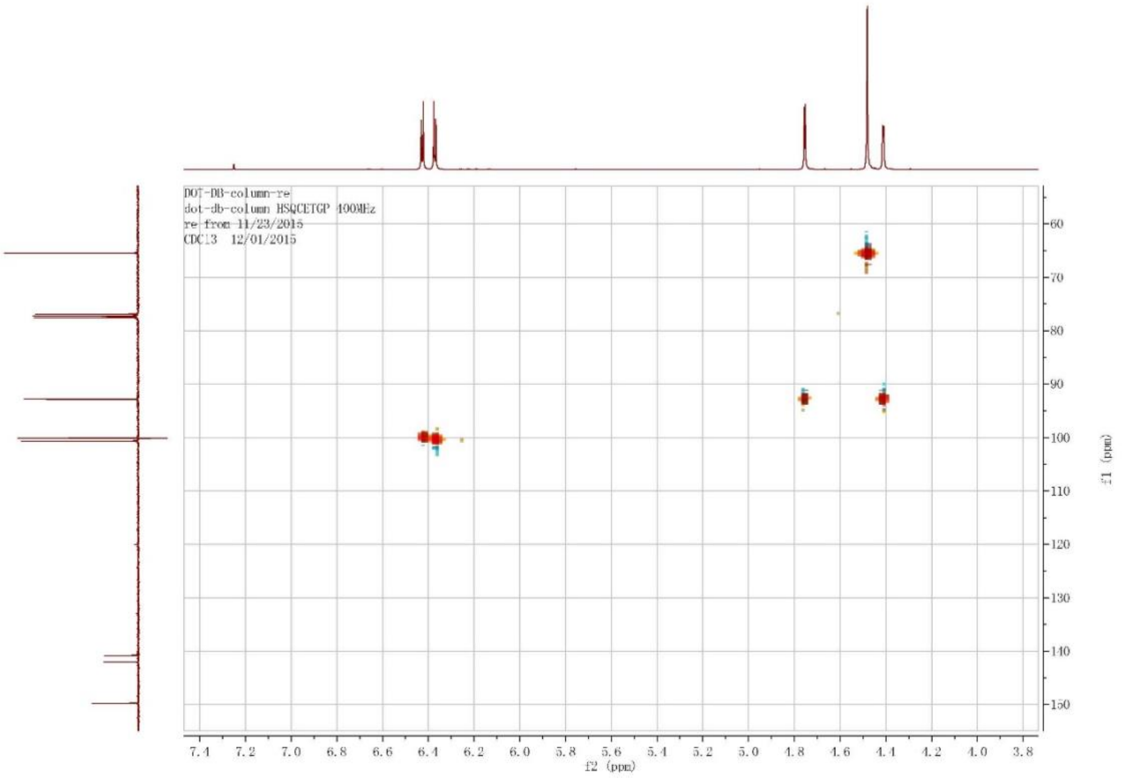


**Figure S3.** 2D HSQC NMR spectra of EDOT-EM

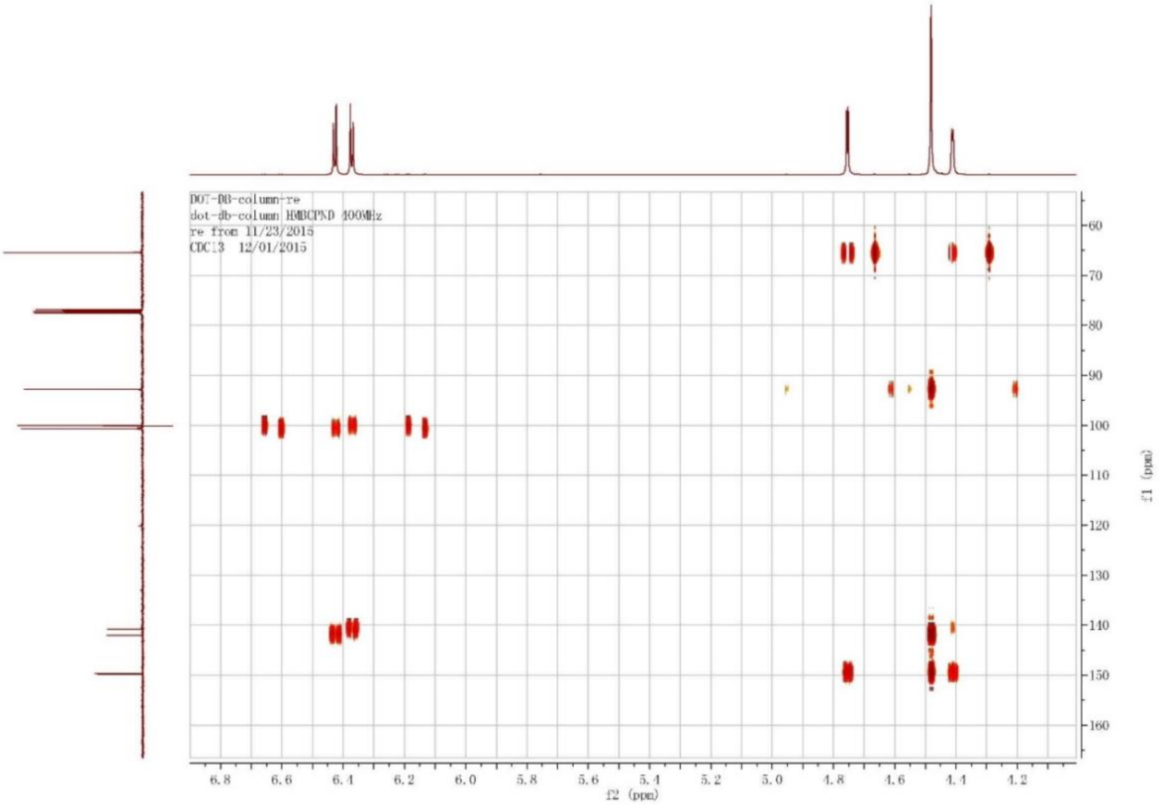


Figure S4. 2D HMBC NMR spectra of EDOT-EM

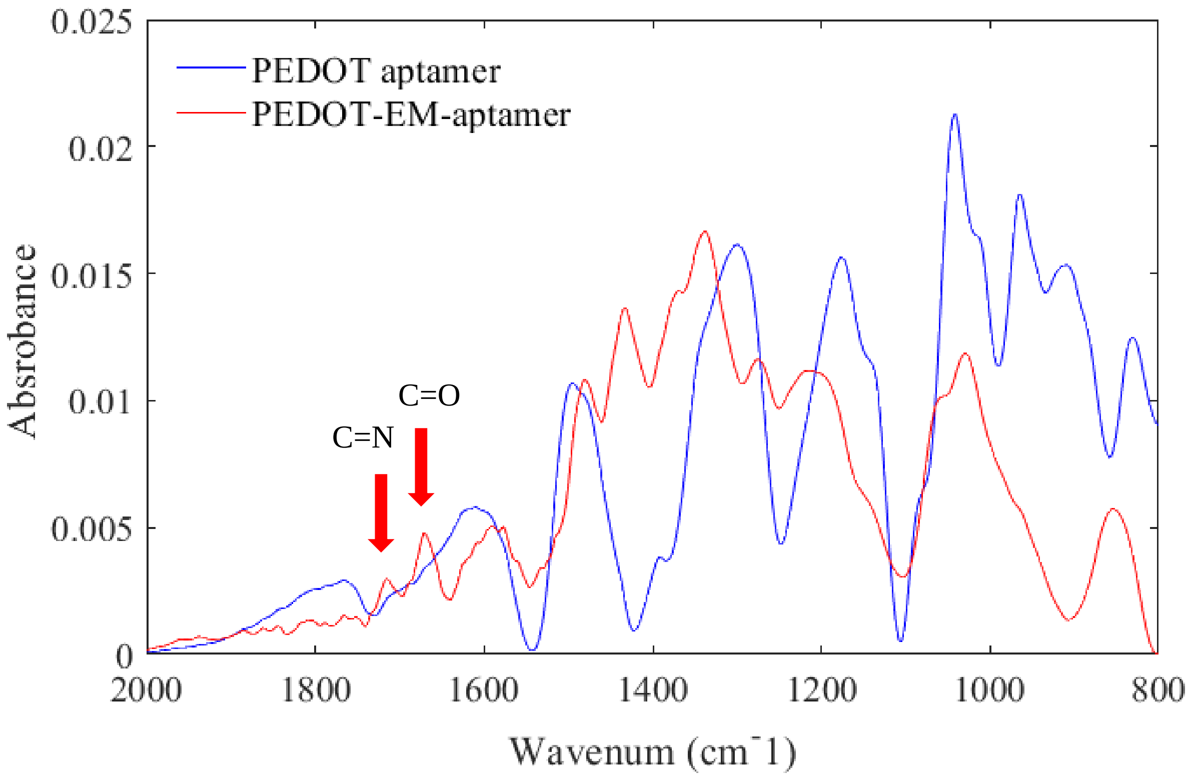


Figure S5 FTIR spectrum comparison of PEDOT and PEDOT-EM after aptamer functionalization. No physical adsorbed aptamer is detected on PEDOT while PEDOT-EM retain the aptamer signature signal, indicated by red arrows. Deposition charge for both films was 249.4mC/cm2.

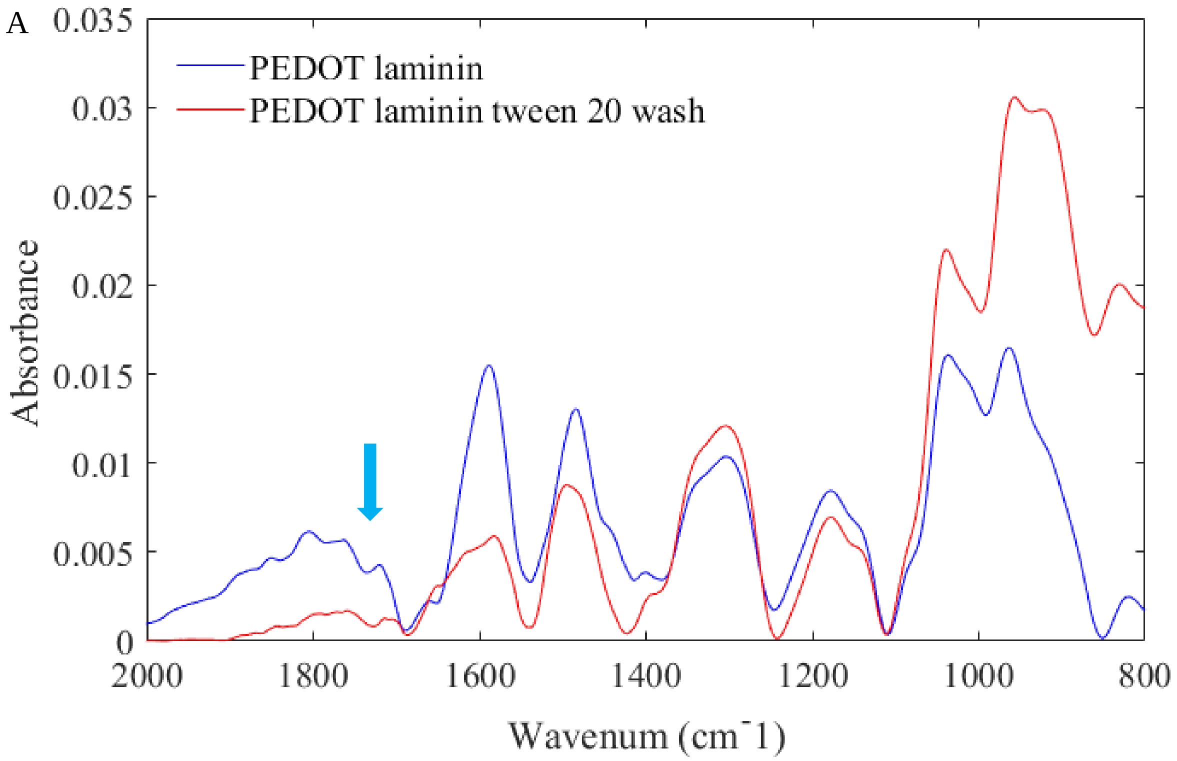


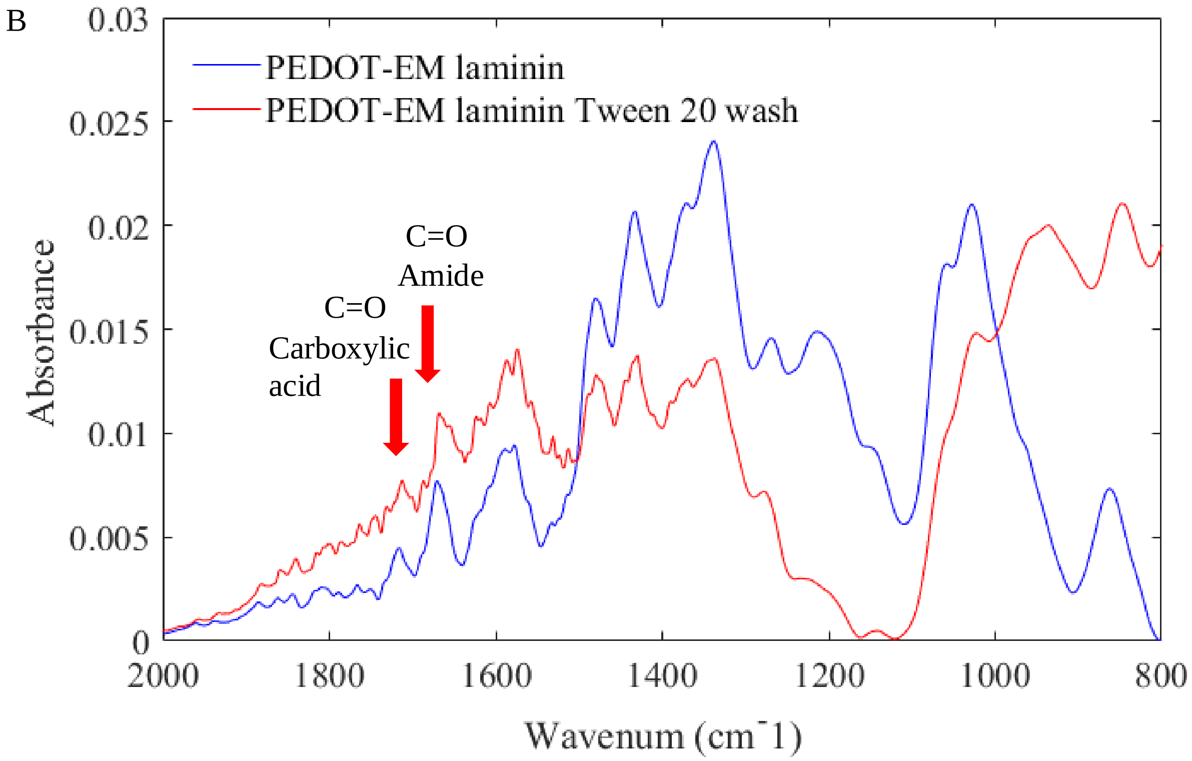


Figure S6. FTIR spectrum comparison of PEDOT (A) and PEDOT-EM (B) treated with Laminin before and after tween 20 wash. Very weak protein signatures are shown for PEDOT-laminin (blue arrow) which disappeared after the tween wash. In the case of PEDOT-EM-laminin, the amide and

carbonyl peaks are more resolved and remain strong after the Tween20 wash. Signatures peaks indicated with arrows. Deposition charge for both coatings was 249.4mC/cm2.
